# Fare to the American Medical Association

**Published:** 1879-05

**Authors:** 


FARE TO THE AMERICAN MEDICAL ASSOCIATION.—For the accommodation of delegates and their families the Great Southern Mail and Kennesaw Route has arranged to place on sale the 26th of April to the 4th of May, inclusive, tickets at reduced rates to Atlanta, Ga., and return, and good until the 20th of May. A Pullman sleeper for delegates from Philadelphia will be placed in position at Broad Street depot (P. W. and B. R. R.) at nine o'clock, P. M., on the 29th of April, at the same hour each evening during the sale of tickets, so that they may avoid loss of rest in waiting for the train, which leaves at one o'clock, A. M. At Washington an additional sleeper will be attached, which will afford ample accommodation. The varied, beautiful, and romantic scenery along this route is unequaled by that along any other, running to the South. Traversing what were the battle fields of Manassas and Bull Run, the train sweeps past the Peaks of Otter, courses through the Roanoke Valley, crosses the Blue Ridge and Alleghany Mountains, winds along the banks of the Tennessee River, and runs in full view of the picturesque mountain ranges of North Georgia, and delivers the passenger in Atlanta, the "Gate City of the South," in less than thirty hours from Washington.

The attention of delegates from Washington, and all points north and east thereof, is called to the requirements necessary to secure reduced rates: application must be made to Dr. W. B. Atkinson, Permanent Secretary of the American Medical Association, No. 1400 Pine Street, Philadelphia, (including stamp), who will furnish certificates, on presentation of which at the various points mentioned the tickets can be purchased to Atlanta and return.

